# Preconcentration and Separation of Mixed-Species Samples Near a Nano-Junction in a Convergent Microchannel

**DOI:** 10.3390/s151229824

**Published:** 2015-12-05

**Authors:** Ping-Hsien Chiu, Chen-Hsun Weng, Ruey-Jen Yang

**Affiliations:** 1 Department of Engineering Science, National Cheng Kung University, Tainan 70101, Taiwan; cyndibegin@gmail.com; 2 Medical Device Innovation Center, National Cheng Kung University, Tainan 70101, Taiwan; b88501113@gmail.com

**Keywords:** microfluidics, preconcentration, ion concentration polarization, electrophoresis

## Abstract

A fluidic microchip incorporating a convergent microchannel and a Nafion-nanoporous membrane is proposed for the preconcentration and separation of multi-species samples on a single platform. In the device, sample preconcentration is achieved by means of the ion concentration polarization effect induced at the micro/nano interface under the application of an external electric field, while species separation is achieved by exploiting the different electrophoretic mobilities of the sample components. The experimental results show that the device is capable of detecting C-reactive protein (CRP) with an initial concentration as low as 9.50 × 10^−6^ mg/L given a sufficient preconcentration time and driving voltage. In addition, it is shown that a mixed-species sample consisting of three negatively-charged components (bovine serum albumin (BSA), tetramethylrhodamine(TAMRA) isothiocyanate-Dextran and fluorescent polymer beads) can be separated and preconcentrated within 20 min given a driving voltage of 100 V across 1 cm microchannel in length. In general, the present results confirm the feasibility of the device for the immunoassay or detection of various multi-species samples under low concentration in the biochemical and biomedical fields. The novel device can therefore improve the detection limit of traditional medical facilities.

## 1. Introduction

In practice, the successful treatment of any disease relies on detecting the onset of the disease at the earliest stage possible. However, when the disease is not fully developed the target biomarker may only be present in very small quantities. Many traditional medical facilities suffer from detection limits. Consequently, existing medical techniques struggle to achieve early disease diagnosis even given the availability of large sample volumes. Thus, effective techniques are required for sample concentration prior to detection in order to enhance the detection sensitivity and improve the accuracy of the detection outcome. For example, the detection of C-reactive protein (CRP) under low concentration is desired. From a pathological standpoint, scientists are interested in knowing whether values in the lower portion of healthy reference interval, possibly representing slight but important cardiovascular disease (CVD)-associated inflammation, are associated with any additional risk for CVD.

CRP is a critical protein generated by the liver cells [1]. When the body is injured, or ischemia causes tissue necrosis, infection or acute inflammation, the concentration of CRP in the blood increases significantly. Consequently, CRP serves as a useful indicator of chronic inflammation or cancer. Moreover, even a tiny increase in the CRP level may point to an increased risk of cardiovascular disease (CVD) since atherosclerosis is a chronic inflammation of the cardiac wall [2]. The clinical value of CRP lies in the fact that it provides a rapid and low-cost means of evaluating tissue damage and acute inflammation.

In performing CRP analyses, clinical applications consider either general CRP or high sensitivity-C-reactive protein (HS-CRP). Studies have shown that inflammation plays an important role in the formation of atherosclerosis [3], and inflammation indicators are certainly the best choice. Understanding of the formation of atherosclerosis indicates that cardiovascular diseases are an inflammatory reaction [1]. In estimating the risk of suffering cardiovascular diseases, HS-CRP is more accurate than all previous indicators, such as low density lipoprotein- cholesterol (LDL-C) [4,5]. A higher level of HS-CRP would lead to a greater risk of suffering cardiovascular diseases. Moreover, it is an independent factor, and free from the influence of high blood pressure, diabetes, smoking, the concentration of serum cholesterol, and family history [6]. Studies have also indicated that those with a low incidence of cardiovascular diseases have a lower level of HS-CRP in the initial stage. Thus, according to the suggestion of the American Heart Association and the Center for Disease Control and Prevention (AHA/CDC) [7], those with moderate conditions of cardiovascular diseases can use HS-CRP as an auxiliary tool to measure the risk of cardiovascular diseases. Therefore, HS-CRP is a highly useful and convenient indicator in clinical medicine. General CRP has a value of <5 mg/L in healthy individuals and is detected with single-digit precision using nephelometry. By contrast, HS-CRP is measured by means of an Enzyme-Linked ImmunoSorbent Assay (ELISA); typically with a sensitivity of around 0.1 mg/L. Many clinical studies have suggested that HS-CRP serves as a useful independent predictor of cardiac infarction, embolic stroke and peripheral arterial disease [6]. Thus, the American Heart Association and Center for Disease Control and Prevention specify the following clinical thresholds for the risk of CVD: HS-CRP < 0.1 mg/dL, low risk; HS-CRP 0.1–0.3 mg/dL, average risk; and HS-CRP > 0.3 mg/dL, high risk [8]. Notably, HS-CRP not only serves as a predictor for the onset of CVD in healthy individuals, but can also be used to quantify the recovery extent of patients post CVD treatment [9,10,11].

As micro-electro-mechanical systems (MEMS) technologies have continued to mature in recent decades, many microfluidic devices have been proposed for detection and diagnosis purposes in the biochemical and biomedical fields. Compared to the traditional macroscale systems used in these fields, microfluidic systems have many advantages, including a lower cost, a greater portability, a lower sample/reagent consumption, a faster throughput, and an improved sensitivity. The literature contains various proposals for sample concentration in such devices, including field-amplified sample stacking [12], isoelectrofocusing [13], temperature gradient focusing [14] and electrodynamic force focusing [15]. However, while such methods are invaluable in improving the sensitivity of the detection process, a problem still remains in that many medical samples actually have the form of multi-species specimens containing both cells and proteins (e.g., blood cells and plasma). Consequently, some form of separation process is still required in order to enhance the reliability of the detection results. While many separation methods are available, electrophoresis-based techniques are the most commonly used. Typical examples include gel electrophoresis [16], capillary electrophoresis [17], isotachophoresis (ITP) [18].

The literature contains many proposals for the immunoassay of samples containing sparse analytes on microfluidic platforms. For example, the authors in [19] presented a method for collecting and analyzing sparse viruses using magnetic nanoparticles. Meanwhile, the authors in [20] proposed a technique for separating biomolecules using a highly-ordered porous gel nanostructure. Shin *et al.* [21] performed the preconcentration of CRP antigen with packed beads in a microfluidic chip to enhance thesensitivity of the fluorescence intensity by 20-fold in the detection system. Ko *et al.* [22,23] demonstrated the potential for achieving CRP preconcentration in a straight microchannel by exploiting the ion concentration polarization (ICP) effect, improving thedetection limit by two orders of magnitude, from 1 μg·mL^−1^ to 10 ng·mL^−1^.

The present study proposes a microfluidic device consisting of a single convergent microchannel and a Nafion-nanomembrane for the simultaneous concentration and separation of mixed biomedical samples. In the device, ion concentration and separation are achieved by applying an external voltage across the microchannel, thereby producing an ICP effect at the micro/nano interface and prompting a separation of the sample components due to their different electrophoretic mobilities. The feasibility of the device is demonstrated by performing CRP detection tests using various sample concentrations and separating a mixed sample consisting of negatively-charged bovine serum albumin (BSA),tetramethylrhodamine(TAMRA) and fluorescent polymer beads.

## 2. Experimental

### 2.1. Materials and Instruments

The main items of experimental apparatus included an optical microscope (Eclipse 50I, Nikon, Tokyo, Japan); two object lenses (4× and 2×), two filter lens (Nikon) with receiving wavelength ranges of 450~490 nm and 510~590 nm, respectively, a CCD camera (SSC-DC50A, Sony, Tokyo, Japan), and a DC power supply (Keithley 2400 source/measure unit). The fluorescence intensity of the captured images was further analyzed quantitatively by integrated optical density arbitrary units (A.U.) using ImagePro Plus software (Media Cybernetics, Silver Spring, MD, USA). The concentration/separation experiments were performed using a buffer solution of phosphate buffered saline (PBS) and tris(hydroxymethy)aminomethane (Tris) with a concentration of 10^−3^ M (pH = 8). We used 0.001 M PBS (0.01 × PBS) ionic strength is 1.627. The PBS was produced by the four compositions including NaCl, KCl, Na_2_HPO_4_ and KH_2_PO_4_. Moreover, the concentration of the fluorescein BSA (Sigma-Aldrich, Saint Louis, MO, USA) was 10^−6^ M (pH = 8) and that of the TAMRA (Sigma-Aldrich) was 10^−6^ M (pH = 8). The fluorescent particles size was 1 μm (Sigma-Aldrich) and had the same excitation wavelength as the BSA solution. The Nafion membrane was prepared using a mixture of Nafion and water diluted to 5 wt%. Finally, an electric potential was applied to the microchip by means of two platinum electrodes

### 2.2. Chip Fabrication

**Figure 1:**
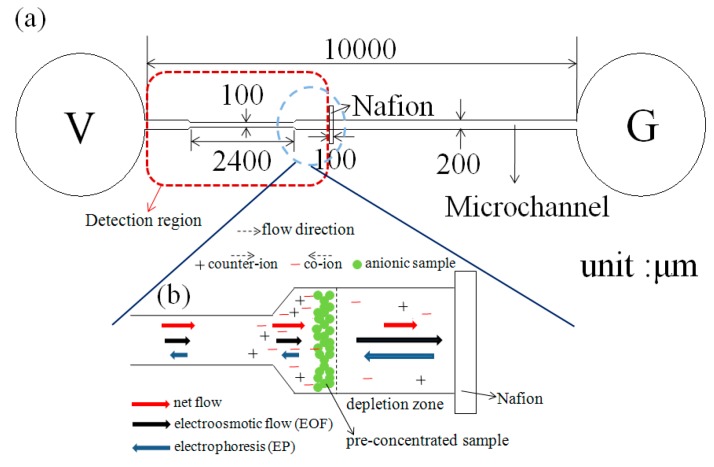
Schematic illustrations of (**a**) Nano-microfluidic chips with a convergent microchannel and (**b**) The flow field, charges and pre-concentrated sample distribution along the microchannel.

Figure 1a shows the nano-microfluidicdevices incorporating a convergent microchannel, and a surface-patterned nano-porous Nafion membrane. The V end of the channel is attached to the anode (supplied with a voltage of 100 V), while the G end is connected to the cathode (grounded). Furthermore, the microchannel is approximately 1 cm long, 200 μm wide, and 20 μm deep. The convergent section of the channel has a width of 100 μm and a length of 2400 μm. Finally the Nafion membrane has a length of 800 μm, a width of 100 μm and a depth of 7 μm.

**Figure 2:**
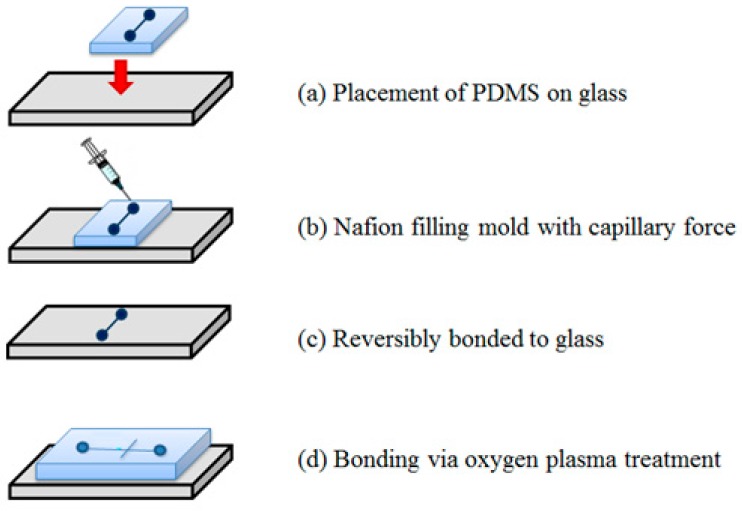
Schematic illustration of fabrication process for pattened Nafion.

The microchannels were patterned on polydimethylsiloxane (PDMS) substrates using a conventional photolithography technique with SU-8 negative photoresist [24]. A second PDMS substrate was then patterned with a microchannel having the same dimensions as the Nafion membrane. Figure 2 shows the process for creating a surface-patterned Nafion junction. The PDMS microchannels (reversibly bonded to glass) were utilized to define the membrane flow path of the resin. First, we put the PDMS microchannels on the glass, injected 5 wt% Nafion at one reservoir, and then, the microchannel was filled via the capillary force. After completely fill the channel and obtain good mixing well, a positive pressure was applied on the filling reservoir. The glass with Nafion resin was placed on a hotplate at 60 °C for 240 min. It is able to fully cure the patterned membrane junction. When we removed the PDMS, a thin layer of membrane can be observed. In the end, the microfluidic chips were completed by sealing the microchannel PDMS with a glass layer by an oxygen plasma treatment. The PDMS chip with microchannel was bonded on top of the glass with a membrane as shown in Figure 2.

### 2.3. CRP Fluorescent-Labeling Protocol

#### 2.3.1. Step 1: Buffer Displacement

The main steps in the CRP sample preparation process are described in the following.

1. Take 200 mM of NaHCO_3_ (600 μL) to Moistenillustra^TM^ MicroSpin^TM^ G-25 Columns (GE Healthcare, Saint Louis, MO, USA)
2. Centrifuge (1 min), and discard the centrifuged waste liquid; repeat twice;
3. Mix CRP (100 μL) with illustra^TM^ MicroSpin^TM^ G-25 Columns (GE Healthcare), centrifuge (2 min), and dissolve centrifuged CRP solution in NaHCO_3_.

#### 2.3.2. Step 2: Cyanine 3 Conjugation

1. Mix 3 μL dimethyl sulfoxide (DMSO) with cyanine 3-NHS ester;
2. Mix 10 μL of solution obtained in third step of Step 1 with 1 μL of solution obtained in first step of Step 2.

Note: Repeat Step 2 three times, mix each Eppendorf once every 15 min for 2 h.

#### 2.3.3. Step 3: Purification

1. Mix the total volume (33 μL) of Eppendorfs obtained in Step 2 with 47 μL of DI water to obtain total volume of 80 μL;
2. Use 600 μL of DI water to moisten newillustra^TM^ MicroSpin^TM^ G-25 Columns (GE Healthcare);
3. Centrifuge (1 min) and then discard the centrifuged waste liquid, repeat twice;
4. Pour illustra^TM^ MicroSpin^TM^ G-25 Columns (GE Healthcare) in, centrifuge (2 min), and obtain centrifuged solution of CRP labeled with cyanine 3-NHS ester.

## 3. Results and Discussion

**Figure 3:**
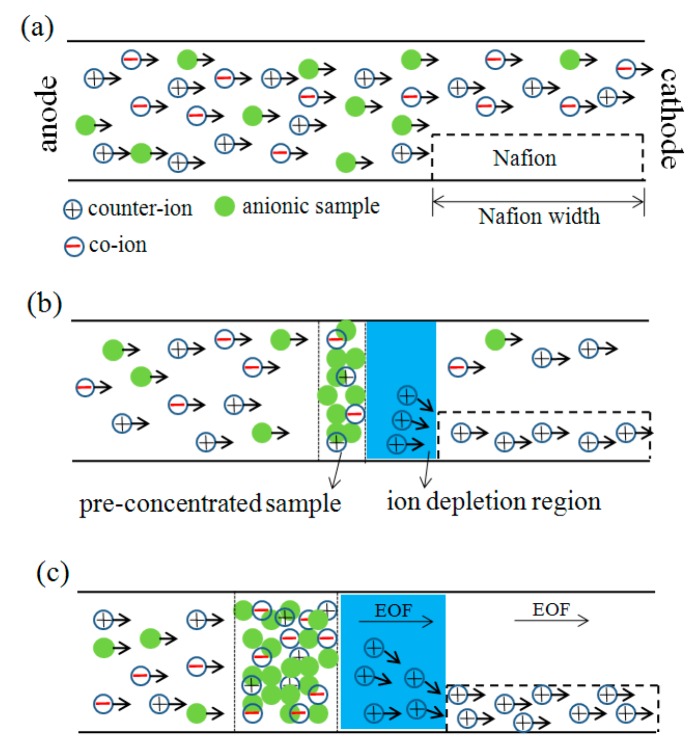
Schematic diagram of ICP and sample accumulation with electroosmotic flow. (**a**) An external electric field is applied across the microchannel; (**b**) ICP effect occurs in which the concentration of the sample molecules gradually increases over time on the left side of the microchannel/Nafion membrane interface; (**c**) The electroosmotic flow carries electroneutral fluid after the depletion region moving above the Nafion membrane continuously to the right direction.

When an external electric field is applied across the microchannel shown in Figure 3a, the combination of electroosmotic flow and migrations of charges makes the net flow to the right direction. Since the Nafion membrane becomes ion-selective due to the formation of double layer overlapping in the nanoporous membrane, a greater number of counter-ions pass through the Nafion membrane than co-ions. As a result, the concentration of co-ions on the left side of the nano-micro interface increases, while that of the counter-ions reduces. In other words, an ionic concentration polarization (ICP) effect occurs in which the concentration of the sample molecules gradually increases over time on the left side of the microchannel/Nafion membrane interface, as illustrated in Figure 3b. As cations continue to pass through the ion depletion regionand migrate into the Nafion membrane by electromigration, anions also move toward the bulk region in order to maintain electroneutrality. As such, the ion depletion region can expand to the left-direction.The electroosmotic flow carries both cations and anions from the left-reservoir, and the anions are trapped or accumulated in the region where the flow is balanced by electromigration force, while the counter-ions migrate through the Nafion membrane. The electroosmotic flow carries electroneutral fluid after the depletion region moving above the Nafion membrane continuously to the right direction, as shown in Figure 3c. If the device has no Nafion membrane, all the above phenomena would not be observed and there is certainly no pre-concentration effect. If the channel depth is filled with 100% Nafion, there is nearly no electroosmotic flow through the Nafion membrane, because the Nafion membrane contains nanopores which can hardly support any significant fluid flow through the microchannel.

**Figure 4:**
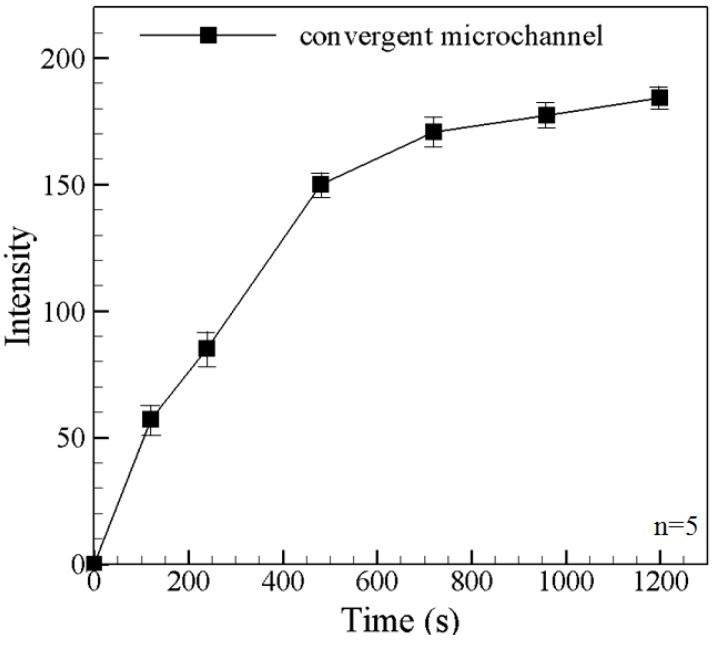
Fluorescence intensity curves of the concentrated BSA under a voltage of 100 V in the nano-microfuidic chips with convergent microchannel. Data points represent the mean of five measurements, and the error bars represent one standard deviation from the mean.

In previous studies [22,25], the ICP effect was induced within a straight microchannel. However, it was shown in [26,27] that a convergent channel results in both a better preconcentration effect and an improved separation performance. Thus, in the present study, the microchannel was also designed with a convergent section on the feed side of the micro-nano interface (Figure 1). The preconcentration performance of the convergent microchannel using a mixed solution of fluorescein BSA with a concentration of 10^−6^ M (pH = 8) and PBS buffer solution with a concentration of 10^−3^ M (pH = 8) under a voltage of 100 V. Figure 4 shows the variation of the measured fluorescence intensity in the microchannel over an observation period of 1200 s. For a preconcentration time of less than approximately 500 s, the fluorescence intensity in the microchannel is nearly increased with time. After 720 s, the concentrated sample moves into the convergent channel. Due to smaller space in the convergent part of the channel, the fluorescein intensity continues to increase [26,27]. Moreover, the intensity continues to increase (albeit more slowly) as the observation time approaches 1200 s as a result of the continuing ICP effect.

**Figure 5:**
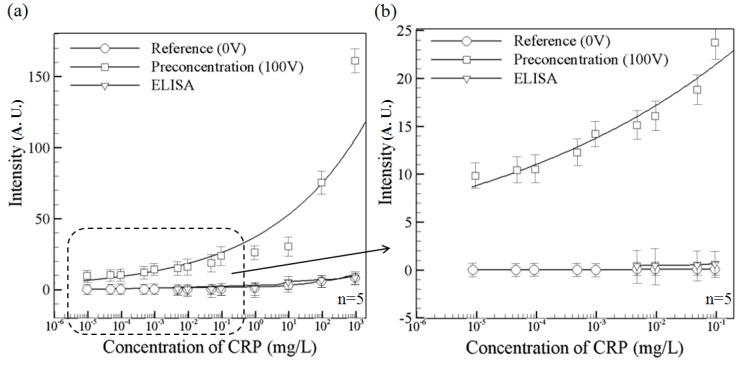
Variation of the concentrated CRP fluorescence intensity with various initial CRP contentrations in the proposed nano-microfluidic chip and ELISA device: (**a**) Intensity variation over full CRP concentration range; and (**b**) Expanded view of intensity variation over partial CRP concentration range. The present chip permits CRP detection to be performed at initial concentrations as low as 9.5 × 10^−6^ mg/L, whilethe ELISA system is unable to detect the concentrations lower than 4.76 × 10^−3^ mg/L. Data points represent the mean of five measurements, and the error bars represent one standard deviation from the mean.

Figure 5a compares the detection performance of the microchip with that of a conventional ELISA system (EZ Read 400, Biochrom, Holliston, MA,USA) using the Human C-Reactive Protein ELISA Kit (Cell Biolabs, Inc. San Diego, CA, USA). Note that the fluorescence intensity data represent themaximum value obtained in the corresponding test. The solid circle line indicates the fluorescence intensity measured in the microchip in the absence of a driving voltage. Meanwhile, the solid square line shows the measured fluorescence intensity given the application of a driving voltage of 100 V. Comparing the two lines, it is seen that for all values of the initial CRP concentration, the ICP effect results in a significant increase in the fluorescence intensity. The enhancement in the fluorescence intensity is particularly apparent in the samples with a higher initial CRP concentration. The solid triangular line shows the fluorescence intensity values obtained by the ELISA system. It is noted that for initial CRP concentrations less than 4.76 × 10^−3^ mg/L, the ELISA system fails to detect the presence of CRP. Note that the reference [28] reported their hospital facility using ELISA system was unable to detect the concentrations lower than 0.1 mg/L, which is about the value we obtained from our ELISA system. Figure 5b presents a magnified view of the fluorescence intensity measurements obtained by the microchip and the ELISA system, respectively. The results confirm that the microchip returns a fluorescence signal at CRP concentrations as low as 9.50 × 10^−6^ mg/L, whereas the ELISA system has a detection limit of 4.76 × 10^−3^ mg/L, a 500-fold improvement is obtained. We determine the signal-to-noise ratio by comparing the measured signals from samples with known low concentrations of analyte with those of containing buffer only. A signal-to-noise ratio between 3 is generally considered acceptable for estimating the detection limit.

**Figure 6:**
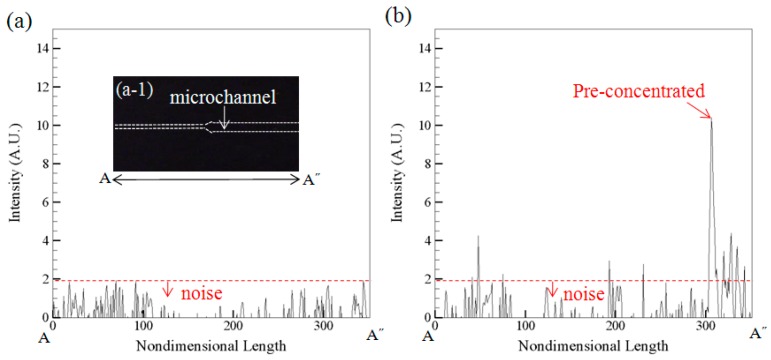
(**a**) The measured intensity along the microchannel A-A˝ when the device contains buffer only; (**b**) The measured intensity along A-A˝ when the device contains both buffer and 9.5 × 10^−6^ mg/L CRP under the pre-concentration operation.

Figure 6a is the measured intensity along the microchannel A-A˝ when the device contains buffer only. Figure 6b is the measured intensity along A-A˝ when the device contains both buffer and 9.5 × 10^−6^ mg/L CRP under the pre-concentration operation. It shows the background value is below 1.9 A.U. which can be regarded as noise (Figure 6a) and the signal value is about 10.6 A.U. (Figure 6b) for pre-concentrated sample under concentration 9.5 × 10^−6^ mg/L.

The signal-to-noise ratio is 5.6. The values were obtained by the “imagepro plus” software. All fluorescence images were converted to 8-bit grayscale and used the “line profile” command to obtain the intensity in A.U. The traditional hospital system used a commercial product “latex-enhanced immunoturbidimetric assay” [28]. The assay needs expensive analytical machine like multi-point calibrators and automated chemistry analyzer. The cost associated with the detection is about ＄10 and it needs 3 h in Taiwan. In our microfluidic chip device, the detection time is 1 h and the cost is about＄3. We hope our developed technique can be widespread to be used in clinical tests. For the current practice, the cost is too high and the detection time is too long. Duration time too long between tests can lead to unreliable results because most of useful analytes may have been metabolized and are no good for further diagnostics. Furthermore, the microfluidic technology offers lower reagent consumption and can beportable for automatic/parallel processing.

**Figure 7:**
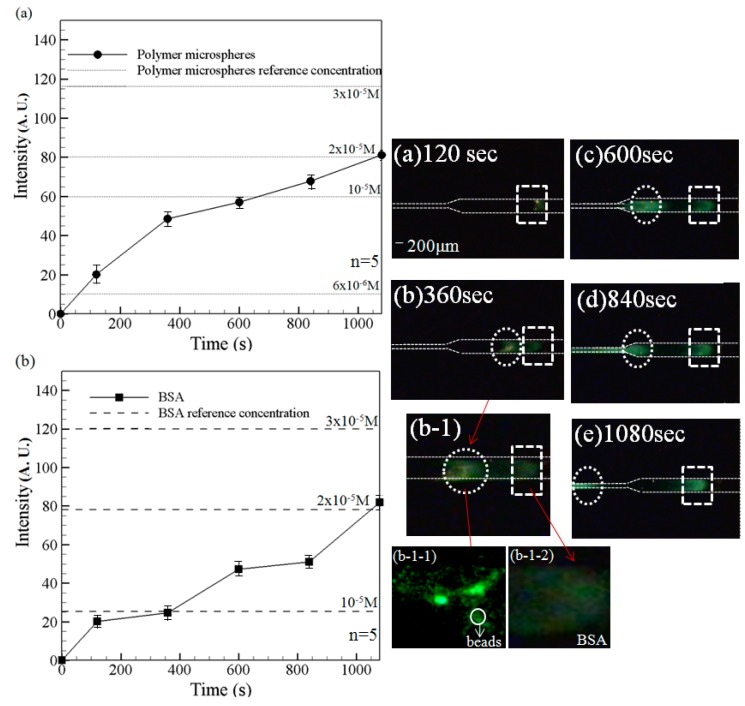
Fluorescence intensity curves of (**a**)polymer microspheres and (**b**) BSA (left) and corresponding experimental images at 120 s, 360 s, 600 s, 840 s and 1080 s (right). Note that driving voltage is 100 V. Figure 7b-1-1: beads aggregation in the left peak; Figure 7b-1-2: BSA aggregation in the right peak. Data points represent the mean of five measurements, and the error bars represent one standard deviation from the mean.

The ability of the microchip to perform the simultaneous preconcentration and separation of a mixed-species sample was investigated using a solution of negatively-charged BSA, TAMRA and fluorescent polymer beads. Note that the sample was prepared using an equal volume (500 μL) of each component. Figure 7 shows the variation over time of the measured fluorescence intensity signals given the use of the filter lens with a receiving wavelength range of 450~490 nm.

It is noted that fluorescence signals are obtained only for the fluorescent particles and the BSA solution since the emission wavelength of TAMRA (510~590 nm) falls outside of the receiving range of the lens. Note also that results are presented for both the preconcentration case (*i.e.*, a driving voltage of 100 V) and the reference case (*i.e.*, no driving voltage). As shown, a notable sample concentration effect occurs over the first 120 s. However, the individual components of the sample cannot be separately discerned (as shown also in the experimental image in Figure 7a). However, as the preconcentration time increases, two separate fluorescence intensity signals are obtained. For example, after 360 s, the experimental images (Figure 7b (2× objective lens) and Figure 7b-1 (4× objective lens)) clearly show both a plug of BSA solution (white dotted rectangular region) and a concentrated region of fluorescent particles (white dotted circular frame). As shown in Figure 7c, corresponding to a time of 600 s, the fluorescence intensity increases for both the fluorescent particles (white dotted circle frame) and the BSA solution (white dotted rectangular frame), indicating a continuing preconcentration effect. Moreover, the separation distance between the two high-fluorescence-intensity regions increases, indicating a sample separation effect. After 840 s, the fluorescence intensity increases for both the particles and the BSA solution. However, of the two samples, the intensity enhancement is particularly apparent for the polymer particles. After 1080 s, the fluorescence intensity of the two species components is very similar (Figure 7e). For both species, the fluorescence intensity is around 85 A.U. In other words, a 20-fold increase in the sample concentration is obtained. In addition, the two species are well separated within the microchannel, as shown in Figure 7e. Thus, the ability of the convergent-microchannel chip to both concentrate and separate multi-species samples is confirmed.

**Figure 8:**
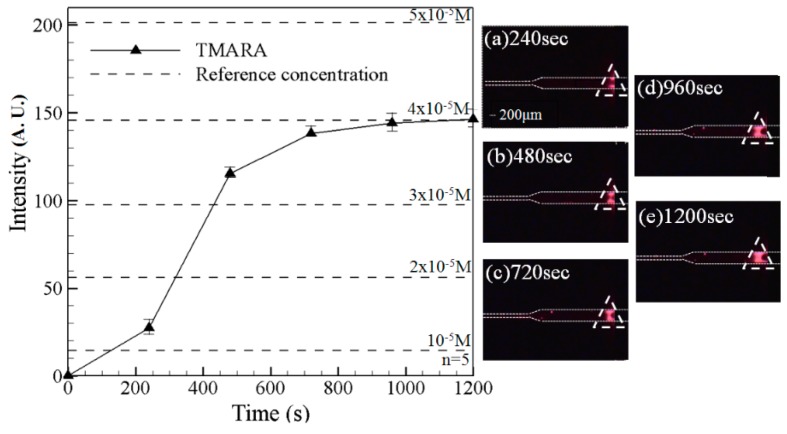
Fluorescence intensity curves of TAMRA (**Left**) and corresponding experimental images at 240 s, 480 s, 720 s, 960 s and 1200 s (**Right**). Note that driving voltage is 100 V. Data points represent the mean of five measurements, and the error bars represent one standard deviation from the mean.

Figure 8 shows the variation over time of the fluorescence intensity signal corresponding to the TAMRA component of the mixed sample. Note that the experimental images were captured using the filter lens with a receiving wavelength range of 510~590 nm. Hence, the fluorescence intensity signals of the BSA (450~490 nm) and polymer beads (450~490 nm) are not observed. Figure 8a shows the formation of a weak-fluorescence-intensity region after 240 s (white dotted triangular frame); indicating a slight preconcentration effect. After 480 s, a noticeable increase in the fluorescence intensity occurs (Figure 8b). A further increase in the fluorescence intensity is also seen after 720 s (Figure 8c); indicating a continuing preconcentration effect. Thereafter, the fluorescence intensity continues to increase; albeit at a slower rate. After 1200 s, the fluorescence intensity is equal to approximately 150 A.U.. In other words, a 40-fold increase in the TAMRA concentration is obtained.

**Figure 9:**
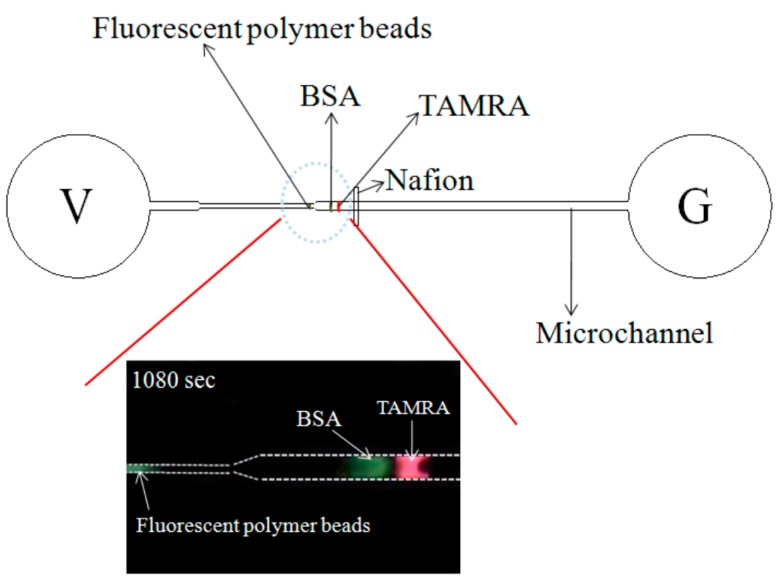
Schematic illustrations of the superimposed image showing the BSA/beads and TAMRA, respectively. It clearly shows the mixed species are both concentrated and separated.

**Figure 10:**
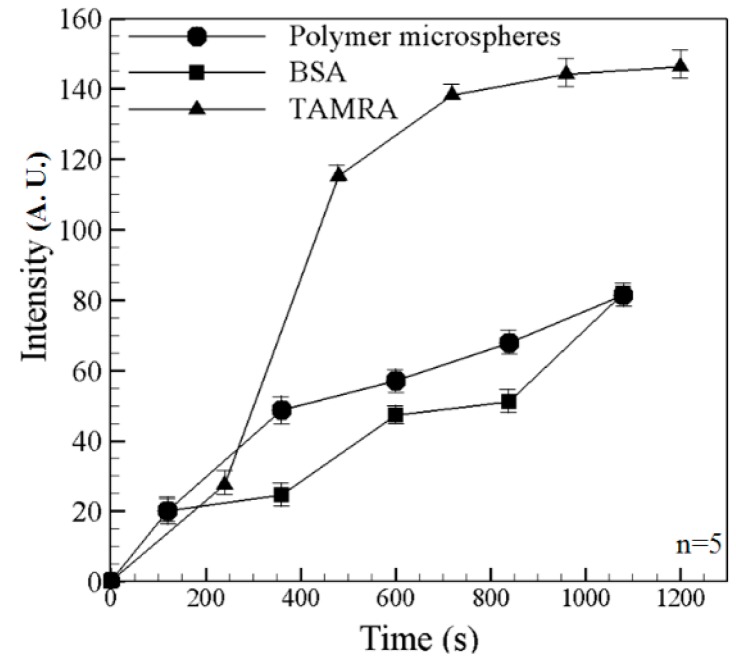
Fluorescence intensity curves of polymer microspheres, BSA and TAMRA at each time point. Data points represent the mean of five measurements, and the error bars represent one standard deviation from the mean.

In obtaining the experimental images shown in Figure 7 and Figure 8, the two filter lens (with receiving wavelengths of 450~490 nm and 510~590 nm, respectively) were switched at intervals of 2 min. In general, the results confirm that the microchip both concentrates and separates the mixed BSA, TAMRA and fluorescent particles. Figure 9 illustrated the superimposed image showing the BSA/beads and TAMRA, respectively, at the final observation time of 1080 s. It clearly shows the mixed species are both concentrated and separated. Figure 10 shows the fluorescence intensity of fluorescent particles, BSA and TAMRA at each time point in Figure 7 and Figure 8. It shows that the fluorescent particles pass through the single convergent channeland the other two (BSA and TAMRA) liquids are not yet into the single convergent channel. As time increases, the fluorescence intensity, the preconcentration intensity and separation distance also increase. Eventually, the BSA and TAMRA will flow into the convergent channel.

## 4. Conclusions

This study has proposed a microfluidic chip consisting of a single convergent microchannel and a nanoporousNafion membrane for performing the simultaneous preconcentration and separation of mixed-species samples. The feasibility of the device has been demonstrated using a dilute CRP solution and a mixed sample of negatively-charged BSA, TAMRA and fluorescent polymer beads. The results have shown that for three samples, a simultaneous concentration and separation effect can be achieved within 1080 s using a driving voltage of 100 V. In addition, it has been shown that the device is capable of detecting CRP in initial concentrations as low as 9.50 × 10^−6^ mg/L. By contrast, traditional hospital detection methods [28] have a CRP detection limit of at least 0.1 mg/L. In other words, the device has significant potential as a low-cost, simple and effective tool for the early detection of CRP; thereby assisting medical staff to identify those at risk of future CVD. The novel device improves the CRP detection limit about 500-fold from using the traditional ELISA facility.
